# Spatiotemporal evolution of the thermal environment and influencing factors in Kashgar City’s Urban Expansion

**DOI:** 10.1371/journal.pone.0341615

**Published:** 2026-02-04

**Authors:** Jiaxing Yan, Wenli Wu, Chunlan Du, Xutong Zhang, Xinyan Sheng

**Affiliations:** 1 Faculty of Agriculture, Shihezi University, Shihezi, China; 2 Faculty of Architecture and Urban Planning, Chongqing University, Chongqing, China; Hunan University, CHINA

## Abstract

Under the dual pressures of global warming and rapid urbanization, the mechanisms governing the evolution of high-temperature environments in arid-zone cities demand systematic analysis. This study investigates the spatiotemporal evolution characteristics and multidimensional influencing factors of urban expansion and thermal environments in the Kashgar region. As a representative city in China’s arid western region, the analysis utilizes multi-source remote sensing data and socioeconomic statistics from 2010 to 2024. Methods including urban expansion indices, surface temperature inversion, and geographic detector models were employed to reveal spatiotemporal patterns linking urban form changes and thermal environments. The findings indicate: (1) Kashgar’s urban expansion exhibits significant spatial heterogeneity, with construction land expansion characterized by stable central areas and rapid peripheral growth. Marginal expansion dominates the thermal environment, where the township-Mulatibag urban-rural fringe has emerged as a new growth pole for thermal expansion. (2) Kashgar’s thermal environment continues to deteriorate, with the area affected by high temperatures increasing by an average of 19.55% annually. The spatial pattern has undergone a three-stage evolution: punctual breakthroughs, cluster aggregation, and contiguous spread. The low-temperature ecological corridor system has systematically shrunk, and the city’s thermal environment regulation capacity shows a significant downward trend. (3) A strong positive correlation exists between Kashgar’s urban expansion and its thermal environment. This correlation exhibited a trend of “initial strengthening followed by weakening” over the fifteen-year period. (4) Factors influencing high temperatures exhibit multidimensional interactions. Among anthropogenic factors, impervious surfaces and population density jointly intensify high-temperature severity through a dual-factor amplification effect. Conversely, natural factors such as vegetation and water bodies exert a negative mitigating influence.

## 1. Introduction

The World Urbanization Prospects Report (2024) states that a thermal environment is a state in which the surface temperature of the environment exceeds the regional climate norm, with significant impacts on human activities and the sustainability of ecosystems. A thermal environment destabilizes ecosystems, constrains economic productivity, increases health risks to populations and exacerbates spatial inequalities in social development [1]. Rapidly expanding urbanization has not only become a gas pedal of changes in urban thermal environments [2–4], but has also further locked the risk of high temperatures in urban spaces [5,6]. As one of the major emerging economies, China’s urbanization rate has increased by 21.65% between 2010 and 2024. Urbanization has led to the expansion of construction land [7,8], thereby exerting significant pressure on urban thermal environments [9,10], and the intensification of thermal environment problems not only deteriorates the quality of urban habitat [11,12], but also puts greater pressure on urban energy and ecological environments [13–15]. Therefore, it is important to study the evolution and influencing factors of the urban thermal environment during urban expansion to create ecology and livability, and the evolution of the urban thermal environment has become one of the hotspots of urban climate and resilience research [16–19].

Research perspectives on urban thermal environments are progressively expanding toward systematic characteristics [20–23]. Esposito, A. et al. [24] emphasized the significant influence of urban form on urban thermal environments, highlighting that morphological analysis is crucial for developing climate adaptation and urban planning strategies; Sarker, T. et al. [25] examined the interdependence between urban expansion and the urban environment by evaluating Jakarta’s spatial patterns over nearly two decades. They revealed a correlation between urban sprawl and worsening thermal environment conditions, while also highlighting characteristics such as built-up areas expanding outward much faster than upward, and urban land density decreasing from the city center to peripheral areas. Han, SS et al. [26] focused their research on the impact of high temperatures on economic spatial patterns and industrial development. They explored how extreme heat drives industries with high cooling demands—such as the information technology sector—to relocate to cooler regions, thereby influencing regional economic restructuring. In summary, the systematic expansion of research on urban thermal environments has encompassed multiple dimensions including urban form, expansion, and economic industries. This not only deepens our understanding of the mechanisms underlying heat impacts but also provides diverse approaches for formulating sustainable urban development strategies.

In urban thermal environment research, methodologies exhibit diverse characteristics, each with specific applications and limitations [27–29]. Cetin, IZ. et al. [30] employed remote sensing inversion techniques to achieve large-scale monitoring of thermal environments in major cities such as Balyan, Turkey. Faiz Alamry et al. [31] employed field measurement methods in the case study of Najaf, Iraq, to construct a governance indicator system incorporating urban green infrastructure. They utilized digital tools to plan a synergistic governance framework for urban development and heatwave management. Wen, D. et al. [32] employed geographical detectors to quantify the differential contributions of core factors such as impervious surfaces and vegetation to regional thermal environments. This approach identified the types of interactions among multiple factors. Moreover, it requires no massive datasets and provides scientific basis for formulating strategies to build climate-resilient cities and mitigate the urban heat island effect.It is evident that diverse research methodologies each have distinct focuses in urban thermal environment studies. They not only adapt to different research contexts to address specific issues but also collectively provide scientific methodological support for building climate-resilient cities and mitigating the urban heat island effect.

In studies examining influencing factors of urban thermal environments, regional characteristics vary across geographic spatial units [33–36]. Hideki Takebayashi et al. [37] found that Tokyo’s spatial average temperature increases due to factors including distance from the coast, industrial facilities, the development of the urban boundary layer, and urban expansion. Iman Rousta et al. [38] identified that influencing factors such as suburbanization, building density, and commercial layout significantly influence Tehran’s urban thermal environment, with the expansion of impervious surfaces and reduced vegetation being primary contributors. These converging findings demonstrate a correlation between urban expansion and urban thermal environments. Baqa, M. F. et al. [39] analyzed Landsat imagery from five distinct years spanning 2000–2020 in the Pakistani port city of Karachi. Using methods such as geogewichted regression analysis, they concluded that the Normalized Difference Building Index (NDBI) is the primary influencing factors in the city’s heat conditions, while scattered vegetation patches in the urban core show no significant correlation with LST.It is evident that regional variations in influencing factors of urban thermal environments are significant, with core driving factors differing across cities. This characteristic provides crucial empirical evidence for developing region-specific heat response strategies.

Under the dual pressures of global warming and rapid urbanization, urban thermal environment management has shifted from isolated technical interventions to a systemic perspective that integrates climate-responsive spatial planning. Said et al. [40] noted in their evaluation of sustainable urban renewal projects that climate resilience serves as the core metric for assessing the effectiveness of urban regeneration initiatives. Renewal plans that neglect climate change often exacerbate thermal environments and degrade ecological buffer functions. Integrating climate adaptation objectives through a multidimensional evaluation framework can significantly enhance a project’s long-term sustainability. This conclusion provides a critical theoretical framework for deciphering the interactive mechanisms between urban spatial changes and thermal environments. Dindar et al. [41] further expanded this perspective through research on urban renewal and sustainable transportation. Their findings reveal that urban expansion patterns directly influence thermal environment evolution: disorderly peripheral expansion exacerbates the heat island effect through contiguous impervious surfaces and increased thermal emissions from transportation. Conversely, synergistic integration of land-use TOD (Transit-Oriented Development) patterns with green corridor-based transportation systems can optimize spatial structures while mitigating thermal environments. This causal logic linking expansion patterns to thermal environment responses offers crucial reference points for addressing thermal environment challenges in policy-driven urban contexts.

Kashgar lies at the junction of China’s Xinjiang region and Central Asia, serving as a quintessential example of an arid city. Its terrain slopes from southwest to northeast, shaped by the unique topography formed by the structural units of the Tarim Basin, the Tianshan Mountains, and the Kunlun Mountain fold belt. The elevation difference between the urban built-up area and its surroundings is remarkably pronounced. With an average elevation of 1,289 meters, the city’s geographical layout features mountains encircling three sides while remaining open on the fourth, with the world’s second-largest shifting desert—the Taklamakan Desert—stretching to the east. Additionally, the region falls within the temperate continental arid climate zone, receiving an annual average precipitation of 59.4 millimeters. Moist air currents from the Indian Ocean and cold currents from the Arctic Ocean struggle to reach the area, contributing to its arid and hot conditions. In recent years, the combination of natural environmental factors and rapid urban expansion has progressively worsened the city’s thermal environment. This paper focuses on Kashgar as a representative arid city to reveal the spatial patterns and influencing factors of urban expansion and thermal environments. By analyzing the evolution of Kashgar’s urban form, the expansion of surface temperatures, and the impact of socioeconomic activities on thermal environments, it constructs an analytical framework applicable to arid cities. This framework provides scientific basis for optimizing urban spatial structures and enhancing urban resilience, while offering insights for sustainable planning and climate-resilient governance and practices in arid cities across Xinjiang, China.

## 2. Materials and Methods

### 2.1. Overview of the study area

**Fig 1:**
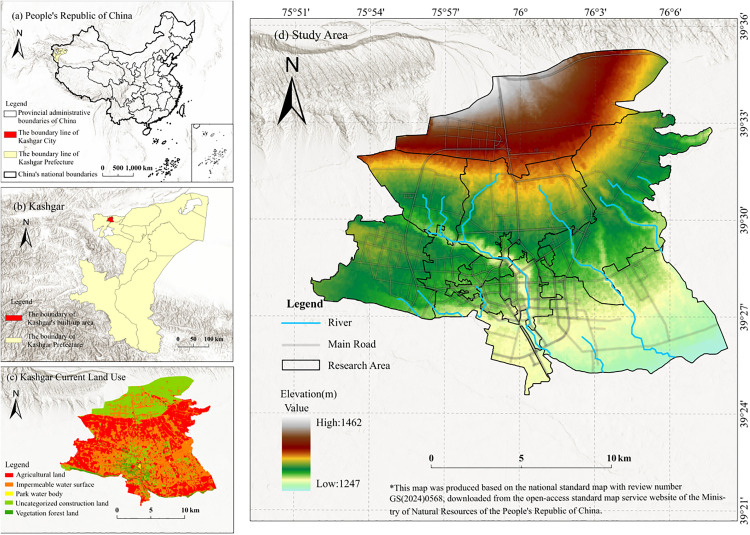
Location map of Kashgar.

Kashgar, situated on China’s western frontier, served as a pivotal junction where the northern and southern routes of the ancient Silk Road converged. As the largest city in southern Xinjiang, its thermal environment evolution mechanism holds significant representativeness and reference value for approximately 15 other small and medium-sized cities in the region sharing similar arid natural geographical conditions. As a national historical and cultural city, its human geography is characterized by multi-ethnic settlement, with the Uyghur population accounting for more than 90% of the population, forming an inter-ethnic mosaic of community patterns and unique cultural landscapes. The total buildable area of the city is 266.38 km², and the population density of the urban area reaches about 3,920 persons/km².The study area is divided into 11 administrative units, including four old city subdistricts (Chassa, Yavagh, Ustangboi, and Kumdelwaza), six urban-rural townships (Dolait Bagh, Hohan, Netser Bagh, Shamal Bagh, Sermon, and Huangdi), and one new township economic development zone (Economic Development Zone).The subdistrict of the old city retain residential houses, historical subdistrict and mosques, and traditional commerce and residential functions are intertwined, with extremely high community density; urban-rural townships have the dual attributes of city and countryside, integrating self-built houses, farmland and scattered commerce nodes, and have become the interactive interface of ethnic cohesion, industrial transfer and urban expansion. On the other hand, the New Town Economic Development Zone is oriented to “industry-city” integration, forming a new type of spatial unit for the linked development of industrial parks, logistics hubs and residential districts, with obvious differences in land use properties (Fig 1).

In May 2010, the Chinese government decided to establish an economic development zone in Kashgar, aiming to build the city into a key gateway for China’s western opening-up through the Belt and Road Initiative. These policies explicitly prioritized the introduction of high-heat-emission industries such as textiles, logistics, and building materials, offering incentives like preferential land prices. By 2017, the construction of numerous projects propelled Kashgar’s urban development into a phase of rapid expansion. By 2024, with the deepening implementation of the Belt and Road Initiative, Kashgar’s urban construction area continued to expand, raising its urbanization rate from 28.53% in 2010 to 50.18% in 2024. This transformation primarily stems from the expansion of construction land during rapid urbanization in China’s arid regions. This expansion has led to a significant increase in impervious surfaces and a marked decline in vegetation coverage, thereby intensifying the spatial differentiation of urban thermal environments. Kashgar’s unique urban spatial pattern and natural geographical characteristics make it a representative case study for examining the evolution of urban thermal environments in arid zones.

### 2.2. Data sources

This study utilizes diverse and abundant data sources. For raster data, Landsat5TM, Landsat7_ETM + , Landsat8_OLI, Landsat9_TIRS-2, and DEM data were obtained from the Geospatial Data Cloud to derive LST, extract surface information, and acquire topographic data. VIIRS/NPP data sourced from the National Oceanic and Atmospheric Administration (NOAA) reflect economic activity intensity. POP world grid data was employed to reflect population density. Vector data included building height information from Tsinghua University’s AI-based national multi-attribute building dataset (CMAB) [42], while POI data was sourced from AutoNavi Maps to represent urban building patterns and human activity intensity. All raster data were uniformly resampled to a 30m spatial resolution and standardized to the same spatial reference coordinate system. Continuous data employed bilinear interpolation, while categorical data utilized the nearest neighbor method.

### 2.3. Research methodology

**Fig 2:**
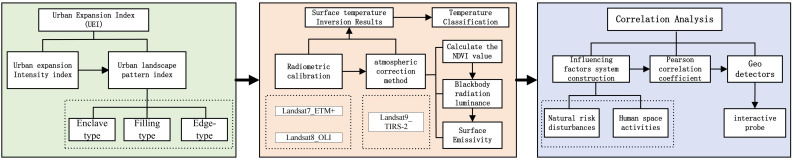
Research Methodology Process.

The study employs a multi-source data fusion and spatial analysis methodology [43–46]: First, Landsat remote sensing data were utilized to perform radiometric calibration, atmospheric correction, and cloud removal for Kashgar City from 2010 to 2024, with maximum likelihood supervised classification employed to interpret land use types. Second, an “urban expansion-thermal environment” analytical framework was established. The Urban Expansion Intensity Index (UEI) and Landscape Expansion Index (LEI) were employed to quantify land development expansion patterns (edge-type, infill-type, enclave-type). Land surface temperature (LST) was derived using atmospheric correction methods, while vegetation coverage was calculated via NDVI to obtain surface emissivity. LST data were then categorized into seven temperature zones. Eleven factors including impervious surfaces (NDISI), water bodies (MNDWI), and population density were selected. Pearson correlation analysis validated the linear relationships between urban expansion and thermal environments. Finally, a geographic detector model analyzed each factor’s explanatory power and interactions with LST, establishing a comprehensive research pathway: “data preprocessing—spatial dynamic quantification—driving mechanism analysis.” (Fig 2).

#### 2.3.1. Calculation of the urban expansion index.

Urban expansion intensity and landscape expansion indices were selected to characterize urban expansion [4, 14]. The urban expansion intensity index is used to express the strength of urban expansion per unit of time:


$$\text{UEI}=\left(\text{A}{\text{t}}_{\text{2}}-\text{A}{\text{t}}_{\text{1}}\right)/\left(\text{A}{\text{t}}_{\text{1}}\times △\text{t}\right)\;\;$$ (1)

In Eq. (1), UEI is the intensity index of urban expansion; $\text{A}{\text{t}}_{\text{1}}$ and $\text{A}{\text{t}}_{\text{2}}$ are the area of urban construction land in the study area in time periods ${\text{t}}_{\text{1}}$ and ${\text{t}}_{\text{2}}$, respectively; and △t is the ${\text{t}}_{\text{1}}-{\text{t}}_{\text{2}}$ interval year.This index represents the average annual growth rate of urban expansion.

The landscape expansion index is used to identify urban expansion patterns, including enclave, infill, and edge patterns. Among them, infill refers to the pattern of expansion within the built-up area; edge refers to the pattern of expansion along the edge of the existing built-up land; and enclave refers to the pattern of expansion where the newly expanded land is not spatially in contact with the existing land:


$$\text{LEI}={\text{L}}_{\text{com}}/{\text{P}}_{\text{new}}$$ (2)

In Eq. (2), LEI  is the landscape expansion index, ${\text{L}}_{\text{com}}$ is the length of the common boundary between the new urban land and the existing urban land; ${\text{P}}_{\text{new}}\;$ is the perimeter of the new urban land; Enclave LEI = 0, Marginal 0< LEI ≤0.5, Infill 0.5<LEI≤1.

#### 2.3.2. Surface temperature inversion.

The primary approach to inverting surface temperature using atmospheric correction is to eliminate the influence of the atmosphere on thermal radiation. The fundamental principle involves quantitatively estimating the radiative effects of the atmosphere through specific methods, subtracting these effects from satellite sensor measurements, and finally performing surface temperature inversion calculations. This process primarily consists of four steps: first, calculating the vegetation coverage within the study area; then, determining the emissivity using empirical formulas; next, calculating the radiant brightness value of a blackbody at the same temperature; and finally, estimating the surface temperature using the inverse of Planck’s equation.

(1) Image Selection and Cloud Removal Processing: Considering Kashgar’s temperate continental climate and its hot, arid summer characteristics, clear-sky images were selected from the three days around August 10th each year, with cloud cover ≤5%. To eliminate the impact of cloud cover on remote sensing analysis, Landsat images underwent preprocessing in the QGIS platform. The core processing method utilized the QA_PIXEL quality band within the imagery. By analyzing the bit flag information of this band, cloud and cloud shadow mask files were generated. Affected pixels were then set to no data and excluded from analysis, ultimately yielding a cloud-free dataset suitable for subsequent analysis.
(2) Vegetation Cover Calculation: Vegetation cover reflects the density of vegetation in an area and serves as a crucial parameter for ecological environment assessment. Its minimum value is 0, and maximum value is 1. The inversion formula is as follows:

$$F_{V}={\left[\frac{\left(NDVI-NDVI_{\text{soil}}\right)}{NDVI_{\text{vegetation}}-\left(NDVI_{\text{soil}}\right)}\right]}^{\text{2}}$$ (3)

In Equation (3): Here, NDVIsoil represents the NDVI value of pure bare soil, while NDVIvegetation denotes the NDVI value of pure vegetation. If the image contains distinct areas of dense vegetation, the average NDVI of this region is used as the value for NDVIvegetation. Similarly, if the image contains distinct areas of bare land, the average NDVI of this region is used as the value for NDVIsoil. Generally, we can set NDVIvegetation to an empirical value of 0.7 and NDVIsoil to an empirical value of 0.05. When NDVI exceeds NDVIvegetation, we can consider it as vegetation-covered area, assigning FV as 1. When NDVI is lower than NDVIsoil, we can consider it as bare soil, water bodies, etc., assigning FV as 0.

(3) Surface Emissivity Calculation: Accurate quantitative measurement of surface emissivity is challenging, and different land cover types exhibit varying emissivities. Typically, land cover is categorized into three types: water bodies, urban areas, and natural surfaces. The corresponding emissivity estimation formulas are as follows:

$$\epsilon_{\text{water}}=\text{0}.\text{995}$$ (4)

$$\epsilon_{\text{surface}}=\text{0}.\text{9625}+\text{0}.\text{0614}{\text{F}}_{\text{V}}-\text{0}.\text{0461}{\text{F}}_{\text{V}}^{\text{2}}$$ (5)

$$\epsilon_{\text{city}}=\text{0}.\text{9589}+\text{0}.\text{086}{\text{F}}_{\text{V}}-\text{0}.\text{0671}{\text{F}}_{\text{V}}^{\text{2}}$$ (6)

In Equations (4), (5), and (6), $\epsilon_{\text{water}}$, $\epsilon_{\text{surface}}$, and $\epsilon_{\text{city}}$ represent the emissivity of water bodies, natural surfaces, and urban pixels, respectively. As shown by the formula, different land cover types correspond to different emissivity calculation methods. We directly estimate the corresponding land surface conditions by utilizing the NDVI values of each pixel in the study area.

(4) Calculation of Blackbody Radiation Brightness Values: The thermal infrared radiation brightness value L received by satellite sensors comprises three components: upward atmospheric radiation brightness L ↑ , the energy from the Earth’s true radiation brightness that reaches the satellite sensor after passing through the atmosphere; and downward atmospheric radiation energy reflected by the ground L ↓ . The expression for the thermal infrared radiation brightness value received by satellite sensors is:

$${\text{L}}_{\lambda }=\left[\epsilon \cdot \text{B}(\text{LST})+(\text{1}-\epsilon ){\text{L}}_{↓}\right]\cdot \tau +\text{L}$$ (7)

In Equation (7), ε represents the surface emissivity, LST denotes the land surface temperature, B(LST) is the thermal radiance of a blackbody at LST, and τ is the atmospheric transmittance in the thermal infrared band. The formula for calculating B(LST) is:


$$\text{B}\left({\text{T}}_{\text{s}}\right)=\frac{\left[\begin{array}{c} {\text{L}}_{\lambda }-\text{L}↑-\tau^{*}(\text{1}-\epsilon )\text{L}↓ \end{array}\right]}{\tau }\tau^{*}\epsilon$$ (8)

(5) Surface Temperature Inversion: The true surface temperature can be obtained using the Planck inverse function, as follows:

$$LST=\frac{K_{\text{2}}}{\text{ln}\left(K_{\text{1}}/B(LST)+\text{1}\right)}$$ (9)

In Equation (9), K1 and K2 are constants. For Landsat 8 thermal infrared band 10, K1 = 774.8853 W/(m²·sr·μm) and K2 = 1321.0789 K (Table 1).

**Table 1:** Parameters for different Landsat sensors.

Sensor type	Wave band	$K_{\text{1}}$ W/(m^2^ ·sr·μm)	${\;K}_{\text{2}}$ (K)
Landsat7_ETM+	Band 6	666.09	1282.71
Landsat8_OLI	Band 10	774.89	1321.08
Landsat9_TIRS-2	Band 10	774.89	1321.08

Note: Due to Kashgar’s arid climate with atmospheric water vapor content < 2g/cm², atmospheric absorption interference on thermal radiation is reduced to some extent. This results in local LST inversion errors 0.5-1.5K lower than those in eastern China’s monsoon cities. Errors gradually decrease with sensor upgrades: Landsat9_TIRS-2 error range is 1.8-3.0K, Landsat 8_OLI error range: 2.0–3.5K Landsat 7_ETM error range: 2.5–4.0K

(6) Surface Temperature Grading: Due to variations in imaging times across different remote sensing images, surface temperature extremes exhibit significant fluctuations and contain outliers, making direct comparative analysis challenging. Therefore, images require normalization processing. This study employs the mean-standard deviation method to classify surface temperatures into seven categories: extremely low, low, relatively low, moderate, relatively high, high, and extremely high. The temperature classification system adopted here references definitions from local climate standards in Xinjiang, ensuring alignment with regional climatic characteristics and enhancing the applicability of research findings for urban planning and practical applications within the region (Table 2).

**Table 2:** Surface temperature class.

Surface temperature class	Criteria for classification
Very low temperature	$\text{LS}{\text{T}}_{\text{i}}<\text{M}-\text{2}.\text{5}\times \text{S}$
Low temperature	$\text{M}-\text{2}.\text{5}\times \text{S}<\text{LS}{\text{T}}_{\text{i}}\leq \text{M}-\text{1}.\text{5}\times \text{S}$
Lower temperature	$\text{M}-\text{1}.\text{5}\times \text{S}<\text{LS}{\text{T}}_{\text{i}}\leq \text{M}-\text{0}.\text{5}\times \text{S}$
Medium temperature	$\text{M}-\text{0}.\text{5}\times \text{S}<\text{LS}{\text{T}}_{\text{i}}\leq \text{M}+\text{0}.\text{5}\times \text{S}$
Higher temperature	$\text{M}+\text{0}.\text{5}\times \text{S}<\text{LS}{\text{T}}_{\text{i}}\leq \text{M}+\text{1}.\text{5}\times \text{S}$
High temperature	$\text{M}+\text{1}.\text{5}\times \text{S}<\text{LS}{\text{T}}_{\text{i}}\leq \text{M}+\text{2}.\text{5}\times \text{S}$
Very high temperature	$\text{LS}{\text{T}}_{\text{i}}>\text{M}+\text{2}.\text{5}\times \text{S}$

Note: LSTi is the LST value of the image i in the study area, M is the LST mean, and S is the LST standard deviation.

#### 2.3.3. Pearson correlation analysis.

The Pearson correlation coefficient is a key measure of the linear relationship between two variables, defined as the ratio of their covariance to the product of their standard deviations [47].


$$r=\frac{\sum_{i=\text{1}}^{N}x_{i}y_{i}}{\sqrt{\sum_{i=\text{1}}^{N}x_{i}^{\text{2}}\cdot \sum_{i=\text{1}}^{N}y_{i}^{\text{2}}}}$$ (10)

Where Xi and Yi represent the ith observed values of variables X and Y, respectively; μX and μY denote the population means of variables X and Y; and n is the sample size. r ∈ [0,1], with a larger absolute value indicating stronger correlation.

#### 2.3.4. Geographical detectors.

Geographical detectors is a statistical method used to analyze the spatial heterogeneity of geographical phenomena and their drivers. It reveals the independent or interactive effects of different factors on geographic phenomena by quantifying the spatial distribution characteristics of geographic elements without the assumption of linearity [48–50].


$$\text{q}=\text{1}-\frac{\sum_{h=\text{1}}^{\text{L}}{\text{N}}_{h}\sigma_{h}^{\text{2}}}{\text{N}\sigma^{\text{2}}}$$ (11)

Where${\;N}_{h}$、$\sigma_{h}^{\text{2}}$ are the sample size and variance of the h th subregion, and $N{,\sigma }_{\;}^{\text{2}}$ are the total sample size and total variance q∈[0,1], with larger values indicating that the factor has more explanatory power for the geographic phenomenon (Table 3). Multiple classification groups were processed using the natural breakpoint method and the equidistant method, respectively (Table 3).

**Table 3:** Geographic detectors interaction types and judgment criteria.

Interaction type	Standard of judgment
Non-linear weakening	$\text{q}\left(X_{\text{1}}\cap X_{\text{2}}\right)<\text{Min}\left(\text{q}\left(X_{\text{1}}\right),\text{q}\left(X_{\text{2}}\right)\right)$
Single-factor nonlinear attenuation	$\text{Min}\left(\text{q}\left({\text{X}}_{\text{1}}\right),\text{q}\left({\text{X}}_{\text{2}}\right)\right)<\text{q}\left({\text{X}}_{\text{1}}\cap {\text{X}}_{\text{2}}\right)<\text{Max}\left(\text{q}\left({\text{X}}_{\text{1}}\right),\text{q}\left({\text{X}}_{\text{2}}\right)\right)$
Two-factor enhancement	$\text{q}\left({\text{X}}_{\text{1}}\cap {\text{X}}_{\text{2}}\right)>\text{Max}\left(\text{q}\left({\text{X}}_{\text{1}}\right),\text{q}\left({\text{X}}_{\text{2}}\right)\right)$
Two-factor nonlinear enhancement	$\text{q}\left({\text{X}}_{\text{1}}\cap {\text{X}}_{\text{2}}\right)=\text{q}\left({\text{X}}_{\text{1}}\right)+\text{q}\left({\text{X}}_{\text{2}}\right)$
Nonlinear enhancement	$\text{q}\left({\text{X}}_{\text{1}}\cap {\text{X}}_{\text{2}}\right)>\text{q}\left({\text{X}}_{\text{1}}\right)+\text{q}\left({\text{X}}_{\text{2}}\right)$

Identify the interaction between the different risk factors ${\text{X}}_{\text{s}}$, assess whether factors ${\text{X}}_{\text{1}}$and ${\text{X}}_{\text{2}}$ increase or decrease the explanatory power of the dependent variant when they act together, or whether the effects of these factors on variant are independent of each other. This is assessed by firstly calculating the q-values of the two factors ${\text{X}}_{\text{1}}$ and ${\text{X}}_{\text{2}}$ on variant: q(${\text{X}}_{\text{1}}$) and q(${\text{X}}_{\text{2}}$) respectively, and secondly calculating the q-value when they interact:q(${\text{X}}_{\text{1}}$ ∩ ${\text{X}}_{\text{2}}$) and comparing q(${\text{X}}_{\text{1}}$), q(${\text{X}}_{\text{2}}$) with q(${\text{X}}_{\text{1}}$ ∩ ${\text{X}}_{\text{2}}$).

Other factors include: employing the Modified Normalized Difference Water Index (MNDWI) to characterize water bodies, utilizing the Normalized Difference Impervious Surface Index (NDISI) to reflect the primary composition of urban surfaces, and applying the Normalized Difference Building Index (NDBI) to represent building density (BD) information. DEM and slope index for terrain representation; corrected nighttime light data for human activity intensity; POP world data for population density distribution; and kernel density analysis of POI data for industrial agglomeration patterns. Each index employs corresponding calculation formulas where spectral band parameters correspond to specific bands in LandsatTM and Landsat8 OLI.

## 3. Results and analysis

### 3.1. Characteristics of the evolution of urban expansion in spatiotemporal terms

From 2010 to 2024, the urban construction land in the study area expanded by a total of 103.5 km². The expansion area and intensity exhibited a pattern of “steady increase in the center and rapid expansion in the periphery,” with significant regional variations. Specifically, the expansion areas in the Economic Development Zone, Dolait Bagh, Hohan, and Shamal were 32.65 km², 29.54 km², 15.40, and 9.118 km², collectively accounting for 74.96% of the total expansion. Their average annual expansion rates were 1.57%, 2.06%, 0.93%, and 2.81%, respectively (Table 4). The evolution of the expansion direction shows the characteristics of “stable center, extending to the east and expanding to the north”, and the old urban areas (Chassa and Yavagh) have the smallest expansion (<0.15 km²), which indicates that the land use in the central urban area tends to be stable. Due to the “Kashgar Urban Master Plan (2011-2020)” policy to promote the development of textile and photovoltaic industrialization in the northern townships, the Economic Development Zone has shown a trend of dramatic expansion of the area; the city’s development across the river, infrastructure investment to drive the expansion of the eastern part of the city, urban-rural townships such as Dolait Bagh have become new growth poles.

**Table 4:** Area and intensity of urban expansion in the study area, 2010–2024.

Number	Name of subdistrict (township)	Expansion area (/km²)	Expansion strength (%/year)
**①**	Chassa	0.107	0.11
**②**	Yavagh	0.062	0.34
**③**	Ustangboi	0.153	0.30
**④**	Kumudelwaza	0.335	0.55
**⑤**	Netser Bagh	8.493	0.81
**⑥**	Shamal Bagh	5.224	0.52
**⑦**	Dolait Bagh	29.537	2.06
**⑧**	Hohan	15.402	0.93
**⑨**	Sermon	9.118	2.81
**⑩**	Huangdi	2.424	0.44
**⑪**	Economic Development Zone	32.646	1.57
**⑫**	Total	103.500	0.94

**Fig 3:**
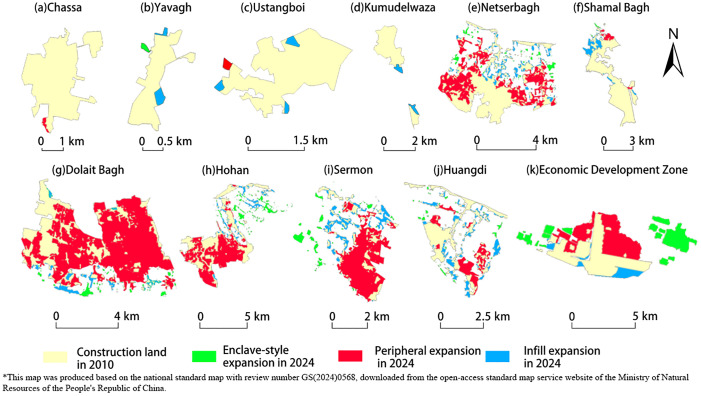
Spatial Distribution of Urban Building Land Expansion Types, 2010-2024.

To eliminate interference from other construction land uses, this study identified urban construction land boundaries based on regional land use plans and analyzed expansion patterns. The predominant expansion pattern across districts and counties was peripheral expansion, with limited instances of infill and enclave expansion. The areas covered by the three expansion patterns were 78.66 km², 17.59 km², and 7.25 km², respectively, with the number of expansion patches being 247, 105, and 34. Peripheral expansion accounted for a significant proportion, exceeding 50% in more than half of the townships. Among them, the proportion of peripheral expansion in urban-rural fringe townships such as Dolait Bage, Shamal Bagh, and Huangdi, where marginal expansion exceeded 80%. Infill expansion areas accounted for a relatively small proportion, primarily concentrated in central old urban districts, with infill expansion in central urban areas reaching approximately 50%. Enclave expansion patches were small in both area and number but widely distributed, accounting for around 5% in most townships. Among these, Netser Bagh and Economic Development Zone had enclave expansion patches representing approximately 20% in both area and number (Fig 3).

### 3.2. Characterization of the spatiotemporal evolution of the urban thermal environment

The high-temperature area of the study area increased by 163.332 km²from 2010 to 2024, with an average annual growth rate of 19.553%, and the annual maximum temperature difference averaged 1.803°C. Among them, it increased by 53.779 km²during 2010–2017 and 109.553 km²during 2017–2024. During 2010–2024, the thermal environment of Kashgar changed significantly. Extremely low temperature and low temperature zone increases first and then disappears, the area expands from 2010–2017 and goes to zero in 2024, then the low temperature zone disappears. The lower and moderate temperature zones continued to shrink, decreasing from 49.216 km² and 131.681 km²to 0.200 km²and 31.271 km², respectively. The higher, high and very high temperature zones expanded significantly. The higher temperature zone increases and then decreases but is still higher than in 2010; the higher temperature zone increases from 14.301 km²to 117.548 km²; and the very high temperature zone increases from none to 40.172 km²in 2024, increasing the high temperature condition in the city (Table 5).

**Table 5:** Area class and percentage of surface temperature from 2010 to 2024.

Level/Years	Very low temperature		Low temperature		Lower temperature		Medium temperature		Higher temperature		High temperature		Very high temperature	
	km²	%	km²	%	km²	%	km²	%	km²	%	km²	%	km²	%
2010	0.119	0.100	2.903	1.100	49.216	18.400	131.400	49.200	69.233	25.900	14.301	5.300	0.000	0.000
2017	0.518	0.200	6.774	2.500	42.171	15.800	80.499	30.100	94.585	35.400	42.502	153.900	0.226	0.100
2024	0.000	0.000	0.000	0.000	0.200	0.100	20.316	7.600	83.922	31.400	122.772	45.900	40.172	15.000

From an administrative perspective, distinct variations in thermal environment evolution are evident across different regions. In Chassa, the area of low-temperature zones expanded from 0.011 km² to 0.178 km² between 2010 and 2017 before declining to 0 km². Meanwhile, high-temperature zones reached 5.863 km² by 2024, reflecting complex changes in the thermal environment. Economic Development Zone witnessed substantial growth in both high-temperature and extreme high-temperature zones. From 2010 to 2024, the high-temperature zone expanded from 14.193 km² to 38.530 km², while the extreme high-temperature zone emerged from nonexistence, reaching 29.553 km² by 2024. This reflects the significant impact of development zone construction on thermal environments.

Some townships also show unique changes, such as the area of the lower temperature zone in Hohan increased from 15.892 km² to 16.850 km² from 2010 to 2017, and decreased to 0 in 2024, and the area of high temperature and very high temperature zones increased significantly after 2017. Specifically, the trend of urban high-temperature enhancement is more prominent with frequent urban industrial and commercial construction activities and the expansion of population and industrial agglomeration to the east and north of the region; while some of Netser Bagh and Hohan, which originally had more low-temperature areas, have seen a rapid decrease in low-temperature areas and deterioration of thermal environments with the passage of time (Table 6).

**Table 6:** Surface temperature class area/km² by administrative region from 2010 to 2024.

Regions/Level		①	②	③	④	⑤	⑥	⑦	⑧	⑨	⑩	⑪	⑫
Very low temperature	2010	0.000	0.000	0.000	0.000	0.000	0.001	0.107	0.000	0.012	0.000	0.000	0.119
	2017	0.000	0.000	0.000	0.000	0.000	0.228	0.289	0.000	0.000	0.000	0.000	0.518
	2024	0.000	0.000	0.000	0.000	0.000	0.000	0.000	0.000	0.000	0.000	0.000	0.000
Low temperature	2010	0.011	0.000	0.000	0.075	0.003	0.565	0.735	0.664	0.849	0.001	0.000	2.903
	2017	0.178	0.000	0.000	0.028	0.006	2.339	1.992	0.528	1.297	0.207	0.000	6.574
	2024	0.000	0.000	0.000	0.000	0.000	0.000	0.000	0.000	0.000	0.000	0.000	0.000
Lower temperature	2010	0.334	0.000	0.000	0.075	6.350	4.470	14.677	15.892	4.619	2.785	0.016	49.216
	2017	0.378	0.000	0.000	0.109	3.074	3.315	8.014	16.850	6.218	4.063	0.049	42.071
	2024	0.000	0.000	0.000	0.000	0.000	0.113	0.000	0.000	0.032	0.029	0.026	0.200
Medium temperature	2010	3.541	0.599	1.644	1.908	22.326	8.976	29.036	24.857	18.489	15.647	4.657	131.681
	2017	0.161	0.059	0.288	0.302	16.302	5.557	13.429	15.030	11.685	12.048	0.937	75.799
	2024	0.286	0.063	0.232	0.449	1.473	1.959	7.097	7.257	3.564	8.777	0.115	31.271
Higher temperature	2010	3.870	0.868	2.329	1.914	4.687	4.681	8.206	4.882	1.313	0.045	36.157	66.869
	2017	6.794	1.406	3.675	3.474	13.286	8.544	26.199	11.706	5.976	3.196	15.328	99.585
	2024	1.292	0.088	0.018	0.653	16.668	4.547	14.959	20.053	14.281	6.324	1.309	80.191
High temperature	2010	0.011	0.000	0.000	0.000	0.021	0.000	0.003	0.069	0.003	0.000	14.193	14.301
	2017	0.137	0.003	0.014	0.000	0.722	0.099	0.748	2.038	0.116	0.096	38.530	42.502
	2024	5.863	1.246	3.536	3.389	13.621	10.948	25.939	16.411	6.376	5.750	24.038	117.548
Very high temperature	2010	0.000	0.000	0.000	0.000	0.000	0.000	0.000	0.000	0.000	0.000	0.000	0.000
	2017	0.000	0.000	0.000	0.000	0.000	0.000	0.000	0.214	0.000	0.000	0.011	0.226
	2024	0.309	0.070	0.190	0.360	1.630	1.150	2.691	2.646	0.677	0.897	29.533	40.172

The core of the city’s thermal environment shows a three-stage evolution of “point breakthrough, cluster aggregation, contiguous spread”: in 2010–2014, the high-temperature patches are concentrated in the subdistrict commercial zone, with a discrete point distribution of single-patch area <0.5 km²; in 2015–2019, with the construction of the International Trade City, a north-south thermal corridor is formed along South Jiefang Road. With the construction of the International Trade City in 2015–2019, the high-temperature pattern forms a north-south thermal corridor along Jiefang South Road, and the core area multiplies; after 2020, the phenomenon of cross-river spreading occurs, penetrating eastward through the three newly built bridges, and the high-temperature front has broken through the ecological barrier of the Tuman River in 2024.

**Fig 4:**
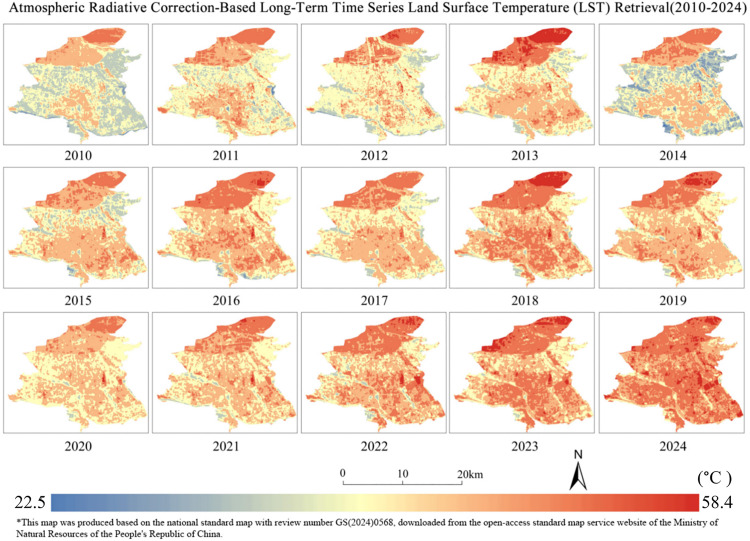
Long-series surface temperature inversion for the study area, 2010-2024.

The cryogenic zone shows the degradation path of “ring defense→island remnant→system decline”: from 2010 to 2012, the cryogenic ecological ring completely wrapped the main urban area, and the two sides of the Tuman River formed a continuous cold corridor with a width of>300m; in 2013, the construction of the western suburb logistics park resulted in the fracture of the western cryogenic zone. In 2017, the subdistrict cold islands in the inner city completely disappeared; by 2024, only 0.38 km² of isolated low-temperature zone in the northern suburb of Wetland Park existed, and its thermal buffering capacity declined. The mapping shows that the evolution of the thermal environment has significant directional heterogeneity: the east-west axis is dominated by the transportation thermal corridor, the north-south axis is coupled with the building density gradient, and the northeast forms a thermally inert zone due to the airport height restriction policy, and this multidirectional difference confirms the spatial differentiation characteristics of the urban thermal environment (Fig 4).

**Fig 5:**
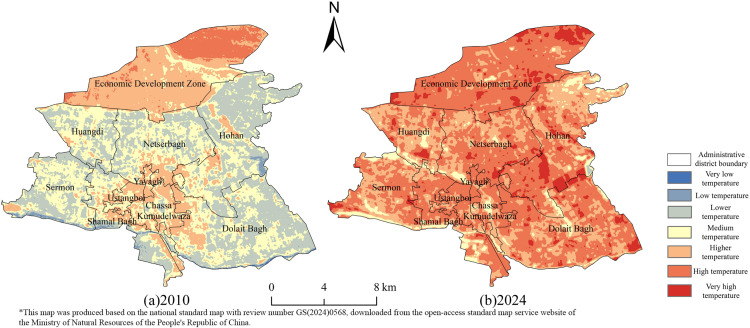
Map of spatial pattern of thermal environment in the study area, 2010 and 2024.

The early high-temperature core area is sporadically distributed around the commercial belt of the old city, and the low-temperature ecological ring forms a continuous barrier through rivers and vegetation. By 2024, the high-temperature zone breaks through the geographic limitations and spreads along the transportation arteries and industrial clusters, forming a heat corridor running through the north and south of the city and expanding to the east across the river. In the 2024 Kashgar thermal environment pattern map, although some areas of the Kashgar Economic Development Zone show local temperature improvement through planning optimization, the overall high-temperature area is still significantly expanding, reflecting the complex game of policy development and high-temperature environment. The low-temperature area is systematically shrinking, with only isolated cold areas remaining, and the fragmentation of river ecological corridors has weakened the natural cooling capacity. In the direction of spatial expansion, high-density development and low vegetation cover in emerging built-up areas exacerbate heat retention, while the temperature rise in traditional high-density urban areas tends to be relatively slower, and there is a threshold effect on the carrying capacity of the urban thermal environment. Unlike the multi-center high-temperature pattern in humid areas, the high-temperature expansion under the special geographic background of arid areas is more irreversible with monoclonal contiguity (Fig 5).

### 3.3. Correlation analysis between urban expansion and thermal environment

In the 2010–2024 study, we constructed an index system of influencing factors of urban expansion and thermal environment based on a variety of data such as Landsat remote sensing data, DEM data, and population density data. First, we quantified the pattern of urban expansion through the urban expansion intensity index and the landscape expansion index, then inverted the surface temperature and divided it into seven temperature classes, and then selected 11 types of factors, such as impermeable surfaces, water bodies, population densities, building heights, etc., from both natural and artificial aspects, so as to analyze their impact on the thermal environment (Table 7).

**Table 7:** Urban thermal environment influencing factors.

Level 1 indicators	Level 2 indicators	Level 3 indicators	Index abbreviation
Natural risk disturbances A1	Thermal risk B1	Surface temperature C1	LST
		Surface energy balance C2	Emissivity
	Vegetation＆Water B2	Vegetation C3	FVC
		Water C4	WNDWI
	Topography B3	Altitude C5	DEM
		Elevation C6	Slope
Human space activities A2	Urban construction intensity B4	Impervious surface C7	NDISI
		Building height C8	BH
		Building density C9	BD
	Socio-economic activities B5	Economic activity C10	EAI
		population density C11	PD
		Industrial point of interest C12	POI

This study employs the proportion of urban construction land in each region during 2010, 2017, and 2024 as independent variables. Utilizing Pearson’s correlation coefficient, it establishes a regression model linking urban construction land expansion with changes in thermal environments, with the proportion of major high-temperature areas serving as the dependent variable. This approach investigates the correlation between urban expansion and Land Surface Temperature (LST). Cases deemed inconsistent with reality or containing errors were excluded. The 2010 regression yielded an R² of 0.477 with a significance level P = 0.048 < 0.05, indicating a strong positive linear correlation. The 2017 regression yielded R² = 0.948, P = 0.007 < 0.01, indicating a highly significant positive linear correlation. The 2024 regression produced R² = 0.640, P = 0.034 < 0.05, showing a strong positive linear correlation.

**Fig 6:**
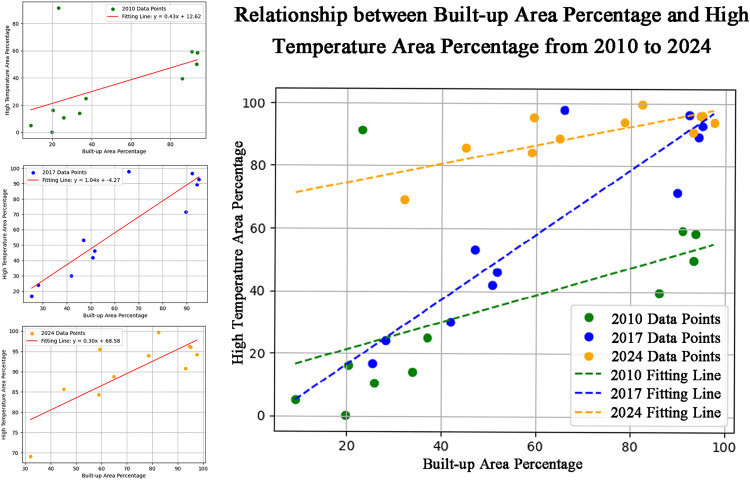
Analysis of correlation between urban expansion and thermal environment.

A closer examination of this correlation over time reveals that the strength of the relationship between the three variables does not exhibit a monotonically increasing or decreasing trend, but rather shows periodic fluctuations. This dynamic pattern suggests that the mechanisms through which urban expansion influences thermal environments vary across different periods, influenced by a combination of factors including development intensity and ecological interventions: the expansion of transportation infrastructure (such as railways and logistics hubs) drives thermal emissions from industrial land along their routes; Regarding development intensity, northern towns concentrated industrialization in high-energy-consuming building material parks, where building density and impervious surfaces synergistically amplified heat; concerning ecological intervention, specialized cooling plans for oasis corridors in arid zones were absent. Unraveling the driving logic behind these phased fluctuations holds significant implications for understanding urban-land interactions in arid regions (Fig 6).

### 3.4. Analysis of influencing factors of urban thermal environment

This study employs a natural segmentation approach to classify 11 factors influencing surface temperature in Kashgar. A geographic detector model is introduced to calculate the explanatory power of each influencing factor on surface temperature (Table 8). Within a reasonable threshold range, the fluctuations in the q-values for all factors remain below 5%, and the p-values for all groups are < 0.005. This indicates that the model exhibits good stability for reclassification thresholds, rendering the analysis results reliable.

**Table 8:** Results of LST Impact Factor Testing.

Influencing factors	Explanatory power q	Reliability p
Emissivity	0.583	< 0.001
FVC	0.647	< 0.001
WNDWI	0.739	< 0.001
DEM	0.344	< 0.005
Slope	0.271	< 0.005
NDISI	0.763	< 0.001
BH	0.535	< 0.001
BD	0.520	< 0.001
EAI	0.396	< 0.005
PD	0.628	< 0.001
POI	0.474	< 0.001

The test results showed that the moderating effects of the factors on the LST showed significant variability. Among the dominant factors, impervious surface (q=0.763) and water body (q=0.739) had the most prominent positive exacerbating and negative mitigating effects on high temperatures, respectively, indicating that urban expansion of hardened surface significantly enhances the intensity of high temperatures, whereas water body is the most effective cooling element; vegetation (q=0.647) and population density (q=0.628) followed closely, confirming that the green space coverage and the population gathering degree had a bidirectional effect on temperature. Secondary factors such as surface energy balance (q=0.583), building height (q=0.535), building density (q=0.520), and industrial activity density (q=0.474) moderated temperature moderately through radiative absorption, spatial shading, and anthropogenic heat emission. Among the weakly correlated factors, the intensity of economic activities (q=0.396) and natural topographic elements (elevation q=0.344, slope q=0.271) had lower explanatory power, reflecting the fact that the thermal environment resulting from urban expansion in Kashgar is more dependent on artificial surface features. The p-values of all factors were 0.00, confirming the statistical significance of the findings.

**Fig 7:**
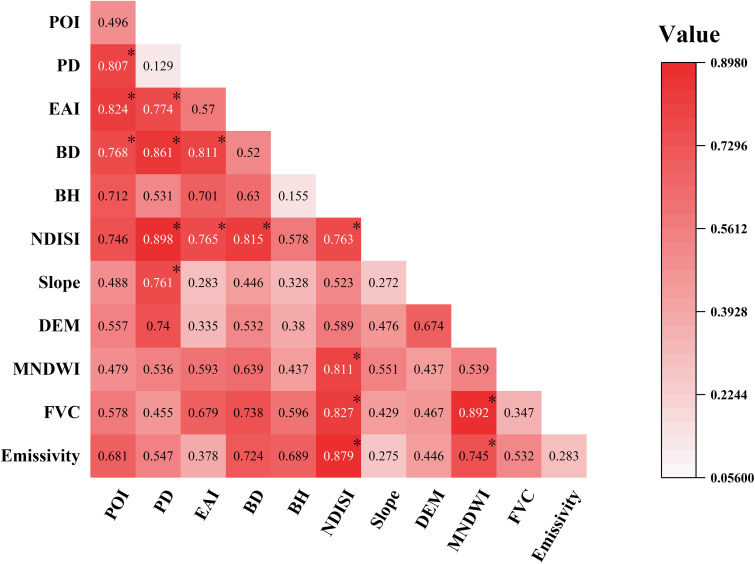
Interaction detection results for each influencing factors. Note: * denotes nonlinear enhancement, unlabeled denotes two-factor enhancement.

The geoprobe analysis reveals the hierarchical differentiation characteristics of the factors influencing the urban high-temperature environment in Kashgar: anthropogenic activities dominate the high-temperature environment expansion through the two-factor enhancement effect, the natural elements show nonlinear enhancement by the resource constraints, and the policy interventions show the special regulation of spatial heterogeneity. For Kashgar, the urban imperviousness faces a very high nonlinear enhancement except for DEM and Slope; the strong interaction between population density and building density (0.898) confirms the positive feedback of “population-buildings” double high density on the core area of the thermal environment, especially in the old city renovation area, which forms the “locking effect” of the thermal environment. The coupling of economic activity and building density (0.861) highlights the strengthening effect of the economic-oriented expansion of industrial parks and logistics hubs on the urban expansion of the thermal environment. In the natural dimension, although vegetation cover and water bodies play a cooling function through nonlinear enhancement (0.892), their efficacy attenuates spatially with distance from the water bodies due to the urban evaporation limitation in the arid zone;The interaction between surface emissivity and urban impervious surfaces (0.879) further reveals that the Tuman River, flowing through the city, forms a strip-like surface radiation barrier due to seasonal water accumulation in the river valley terrain. This barrier exhibits a segmenting effect on the thermal environment. The cutting effect of impervious surfaces (q = 0.763) and water bodies (q = 0.739) were most prominent in positively exacerbating and negatively mitigating high temperatures, respectively (Fig 7).

Generally speaking, in the evolution of the thermal environment in Kashgar, the geographic and climatic background constitutes an innate constraint. The terrain is surrounded by mountain ranges, and the dry and hot air flow from the eastern desert is blocked by the terrain, forming a static wind environment, making it difficult for heat to spread; the natural vegetation is sparse under the extreme arid climate, and the evaporation capacity of the surface is extremely weak, so the natural heat buffer function is nearly absent. Such geographic and climatic conditions are superimposed on urban expansion, forming a composite thermal environment of “topography-climate-impermeable surface”.

The accumulation of high temperatures is exacerbated by policy-oriented industrialization and spatial expansion patterns. The “Belt and Road” promotes the centralized layout of steel manufacturing and textile processing industries with high heat emissions in the northern economic development zone, releasing a large amount of heat from the production process; urban expansion is characterized by “stability in the center and dramatic growth in the periphery”, and the impervious surfaces of urban-rural townships in the east are rapidly expanding. The rapid expansion of urban expansion is characterized by a “stable center and dramatic increase in the periphery”, and the impervious surface of the eastern townships combining urban and rural areas is rapidly expanding, and the population density is very much increased, forming a contiguous heat storage area with the industrial heat sources. This development pattern makes the artificial heat source and the hardened surface grow simultaneously, which strengthens the spatial diffusion of the heat island effect.

The interaction between man-made and natural elements is not reciprocal. As the impermeable surface increases the surface heat accumulation capacity, the duration of the heat island is prolonged at night; the industrial production and transportation emissions in densely populated areas further release heat, and the two synergistically amplify the intensity of high temperatures. The cooling effect of vegetation transpiration and water evaporation along the Tuman River is greatly reduced in the arid environment, which cannot effectively mitigate the impact of artificial heat sources, and ultimately leads to the continuous deterioration of the thermal environment. This imbalance of “strong drive - weak mitigation” is a result of the special topographic and climatic conditions of Kashgar, the policy-guided urban development mode, and the natural thermal buffer function shows obvious insufficiency, which makes the evolution of the thermal environment in Kashgar more complicated than that of other cities in arid zones.

## 4. Discussion

Against the backdrop of global warming and accelerated urbanization, Kashgar—a typical arid city—exhibits distinct differences from cities studied in existing international research. Here, impervious surfaces and population density jointly serve as the dominant factors influencing its thermal environment. Baqa, M. F. et al.‘s [39] study on Karachi, Pakistan, indicates that the normalized building index is the primary factor affecting high-temperature conditions during urban expansion in arid regions. Unlike Karachi, however, Kashgar’s topography—surrounded by mountains on three sides—further impedes the dispersion of urban thermal environment. This geographical constraint renders the “vegetation transpiration mitigating urban thermal environment” mechanism observed by Yin, Z. et al. [51] in New York ineffective in Kashgar, where annual precipitation averages only 59.4 mm. Consequently, vegetation’s cooling efficacy is less than half that of humid regions. Kashgar’s peripheral expansion has led to a surge in impervious surfaces and population density, displacing native vegetation and farmland with construction land. This has significantly altered the surface energy balance, creating a persistent thermal environment.

Empirical analysis of Kashgar unequivocally supports the prevailing consensus within the international academic community: a significant positive correlation exists between urban expansion patterns and the deterioration of thermal environments. This finding corroborates the conclusion proposed by Georgescu, M. et al. [52] that “urban expansion is a key driver of worsening thermal environments,” indicating that despite variations in urban location and developmental stage, the negative thermal environmental effects induced by urbanization exhibit cross-regional universality. As a representative case study of arid-zone cities, Kashgar’s specific spatial form—dominated by “peripheral expansion”—closely couples with human factors such as the rapid increase in impervious surfaces, accelerated population density growth, and significant reduction in natural vegetation. These elements collectively drive the formation and spatial spread of thermal environments. These findings not only provide mechanistic validation for the international research framework positing “urban spatial form as a key determinant of thermal environments,” but also highlight that within the unique natural-geographical context of arid regions, this positive correlation manifests with particular intensity and exhibits more complex underlying mechanisms.

The analytical framework and methodological system developed in this study demonstrate strong applicability and transferability to small and medium-sized cities in southern Xinjiang with similar natural geographical conditions. Most cities in southern Xinjiang are located on the periphery of the Tarim Basin, sharing common characteristics such as arid conditions with low rainfall, sparse surface vegetation, significant diurnal temperature variations, and urban expansion constrained by water resources and topography. The formation and evolution mechanisms of their thermal environments exhibit high structural similarity to those of Kashgar. The research pathway—based on remote sensing inversion, urban sprawl indices, and geographic detectors—that examines “urban heat data—urban sprawl patterns—factors influencing high temperatures” can systematically reveal the common patterns of thermal environment evolution in such cities during rapid urbanization. Preliminary statistics indicate that the models and methods developed in this study are applicable to small and medium-sized cities in Southern Xinjiang, which similarly face high-temperature risks. Future work can extend this analytical framework to assess and spatially regulate thermal environments across the entire arid urban cluster of Southern Xinjiang through parameter localization and threshold calibration, providing a comparable methodological foundation for regional-scale climate adaptation planning.

Overall, the Kashgar case reveals a unique logic in the evolution of thermal environments in arid-zone cities: peripheral expansion and policy-driven growth form a “dual engine” for heat generation, with artificial surface characteristics exerting a far greater regulatory effect on temperature than natural endowments. Ecological restoration experiences from humid regions face significant regional adaptability challenges here. This finding not only deepens our understanding of thermal environments in arid cities but also offers crucial insights for similar cities along the Belt and Road Initiative. Their thermal environment governance must transcend traditional paradigms by integrating arid climate adaptation strategies into industrial layout, spatial form, and ecological conservation. Future research could further combine climate resilience assessments to explore the spatial linkage mechanisms between thermal risks and social equity, providing more comprehensive scientific support for sustainable urban planning.

## 5. Conclusion

This study analyzes the characteristics and influencing factors of the evolution of urban expansion and the thermal environment in Kashgar from 2010 to 2024.The key findings are as follows:

(1) Urban expansion exhibits significant spatial heterogeneity: Construction land expansion in Kashgar City shows stable central growth and rapid peripheral expansion, concentrated in emerging growth poles such as Dolait Bagh and Economic Development Zone. Marginal expansion dominates the spatial differentiation of the city’s thermal environment, with infill development prevailing in old urban districts and enclave expansion accounting for a low proportion.
(2) Kashgar’s urban thermal environment has continuously deteriorated over the past 15 years. Results indicate a substantial increase in thermal areas and a significant rise in the proportion of thermal zones during the study period. The spatial pattern of thermal environments underwent a three-stage evolution: punctual breakthroughs, cluster aggregation, and contiguous spread. Conversely, low-temperature zones experienced a gradual decline, resulting in a significant reduction in the city’s thermal buffering capacity. Further analysis reveals that Economic Development Zone accelerate the formation of high-temperature cores through intensive development, while the urban-rural fringe area of Dolait Bagh has emerged as a new growth pole for worsening thermal environments.
(3) Urban expansion exhibits a strong positive correlation with thermal environments. The expansion of construction land serves as the core driver intensifying urban thermal environments, with this correlation showing a trend of “initial strengthening followed by weakening” over the 15-year period.
(4) The influencing factors of the thermal environment are characterized by multi-dimensional interactions. Among the anthropogenic factors, impervious surface and population density dominate the intensity of high temperature through the two-factor enhancement effect, and their explanatory power is significantly higher than that of the natural factors; although the vegetation cover and the water body have the function of cooling down the temperature, their mitigation efficacy gradually declines with the urban expansion due to the resource endowment of arid zones. Although the river valley effect forms a local thermal environment barrier, the low-temperature ecological corridor is gradually disintegrated by the impact of rapid urbanization.
